# Use of the wetting method on cassava flour in three konzo villages in Mozambique reduces cyanide intake and may prevent konzo in future droughts

**DOI:** 10.1002/fsn3.317

**Published:** 2015-11-20

**Authors:** Dulce Nhassico, James Howard Bradbury, Julie Cliff, Rita Majonda, Constantino Cuambe, Ian C. Denton, Matthew P. Foster, Arlinda Martins, Adelaide Cumbane, Luis Sitoe, Joao Pedro, Humberto Muquingue

**Affiliations:** ^1^ Department of BiochemistryFaculdade de MedicinaUniversidade Eduardo MondlaneMaputoMozambique; ^2^ EEGResearch School of BiologyAustralian National UniversityCanberraACT2601Australia; ^3^ Department of Community HealthFaculdade de MedicinaUnversidade Eduardo MondlaneMaputoMozambique; ^4^ Instituto de Investigacao Agraria de Mocambique (IIAM)NampulaMozambique; ^5^ Direccao Provincial de SaudeNampulaMozambique

**Keywords:** Cassava flour, cyanide, konzo, urinary thiocyanate, wetting method

## Abstract

Konzo is an irreversible paralysis of the legs that occurs mainly in children and young women associated with large cyanide intake from bitter cassava coupled with malnutrition. In East Africa outbreaks occur during drought, when cassava plants produce much more cyanogens than normal. A wetting method that removes cyanogens from cassava flour was taught to the women of three konzo villages in Mozambique, to prevent sporadic konzo and konzo outbreaks in the next drought. The intervention was in three villages with 72 konzo cases and mean konzo prevalence of 1.2%. The percentage of children with high (>350 μmol/L) urinary thiocyanate content and at risk of contracting konzo in Cava, Acordos de Lusaka, and Mujocojo reduced from 52, 10, and 6 at baseline to 17, 0, and 4 at conclusion of the intervention. Cassava flour showed large reductions in total cyanide over the intervention. The percentage of households using the wetting method was 30–40% in Acordos de Lusaka and Mujocojo and less in Cava. If the wetting method is used extensively by households during drought it should prevent konzo outbreaks and chronic cyanide intoxication. We recommend that the wetting method be taught in all konzo areas in East Africa.

## Introduction

The wetting method is a simple method of removing residual cyanogens (mainly linamarin and acetone cyanohydrin) from cassava flour. The method involves mixing cassava flour with water and then leaving the wet mixture in a thin layer for 2 h in the sun or 5 h in the shade for hydrogen cyanide gas produced by the enzymatic and nonenzymatic breakdown of cyanogens to be evolved (Bradbury 2006; Cumbana et al. 2007; Bradbury and Denton 2010). The wetting method was field‐tested in Mozambique in 2005 (Muquingue et al. 2005; Nhassico et al. 2008) and was found to be easy to use and popular with rural women. The thick porridge (nchima) produced by boiling the cassava flour in the traditional way had lost the bitter flavor, due to residual bitter linamarin (King and Bradbury 1995) remaining in nchima made from flour not treated by the wetting method. The wetting method was also taught to village women in southern Tanzania where there had previously been an outbreak of konzo (Mlingi et al. 2011).

Konzo is a spastic paraparesis that causes irreversible paralysis of the legs, mainly among children and young women. It is associated with a high intake of cyanide from a restricted diet of bitter (high cyanide) cassava combined with malnutrition (Nzwalo and Cliff 2011; Cliff et al. 1985; Howlett et al. 1990; Banea et al. 2015a). Konzo epidemics occur at times of agricultural crisis, such as during drought or war in the poorest rural cassava‐staple areas of Africa. Konzo occurs in the Democratic Republic of Congo (DRC), Mozambique, Tanzania, Cameroon, Central African Republic, and Angola. Konzo is a persistent public health problem in the DRC, occurring in at least four provinces and with rapidly increasing incidence in Bandundu Province (Banea et al. 2015b). A recent study in a konzo area found that children with and without konzo had impaired neurocognition compared with controls from a non‐konzo area (Boivin et al. 2013). In Mozambique, children in konzo areas have high urinary thiocyanate levels, a measure of their cyanide intake over previous days, at the time of the cassava harvest (August–October). Apparently healthy school children in these areas, have also shown signs of subclinical neurological damage (Ernesto et al. 2002).

In 2010 the wetting method was taught to the women of Kay Kalenge village in Bandundu Province of DRC where there were 34 konzo cases, and they used it daily to remove cyanogens from their cassava flour. No new cases of konzo occurred during the 18 month intervention, the cyanide content of the treated cassava flour reduced to below 10 ppm and the urinary thiocyanate content of the school children, reduced to safe levels below 350 μmol/L (Banea et al. 2012) Konzo had been prevented in Kay Kalenge by the regular use by the village women of the wetting method, which greatly reduced their cyanide intake. Fourteen months after the intervention ceased in Kay Kalenge we found that the women were still using the wetting method, urinary thiocyanate levels in school children were still below 350 μmol/L, there were no new cases of konzo and the wetting method had spread by word of mouth to three adjacent villages (Banea et al. 2014a) The wetting method is still being used there 5 years later. Subsequently, there have been three more interventions to control konzo in DRC and konzo has now been prevented in 13 villages with a total population of nearly 10,000 people (Banea et al. 2013, 2014b, 2015a). The wetting method has recently been recognized by the World Bank, FAO and WHO as a “sensitive intervention” to remove cyanogens from cassava flour.

In Mozambique and Tanzania epidemics of konzo have occurred due to drought, when the cassava plant is stressed and makes 2–4 times the normal amount of linamarin, the major cyanogen of cassava roots and leaves (Bokanga et al. 1994; Cardoso et al. 2005). During drought the cyanogen content of cassava flour is greatly increased, (Cardoso et al. 2005) village people get sick from cyanide poisoning (acute cyanide intoxication) and many change their processing method from sun drying to heap fermentation, which reduces the cyanide content of flour by about 50% (Ernesto et al. 2002). However, this reduction is insufficient to prevent the occurrence of konzo and ongoing chronic cyanide intoxication during a drought (Cardoso et al. 2005) and the wetting method is therefore needed as an additional processing method to greatly reduce cyanide intake.

In this study, we describe the introduction of the wetting method in three villages in Nampula Province of Mozambique in which there are many konzo cases from previous droughts and/or war, but few sporadic cases in recent years, and we compare these results with the situation in konzo villages in the DRC, where konzo is occurring in increasing numbers every year.

## Experimental

### Study area and sample collection

**Figure 1:**
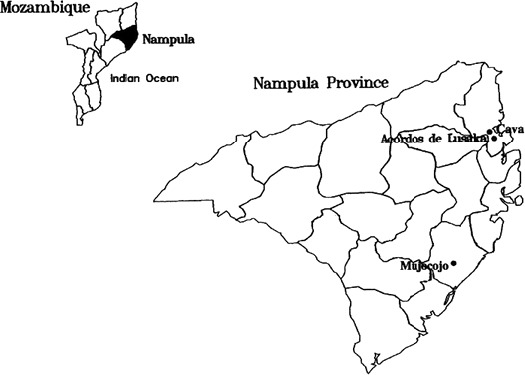
Map of Nampula Province showing district boundaries and the location of the three villages. The inset map shows the location of Nampula Province within Mozambique.

In September 2012 representatives of the research teams met with health, administrative authorities and community leaders to identify three villages in Nampula Province which were most affected by konzo. The three villages were Cava, and Acordos de Lusaka (also called Miaja) in Memba District and Mujocojo in Mogincual District, see Figure 1. In Memba, konzo epidemics have been associated with drought, beginning with a severe drought in 1981. In Mogincual, a large epidemic was associated with war in 1992–3. Both Cava and Mujocojo have continued to record sporadic cases, with Cava suffering an epidemic due to drought in 2005. Acordos de Lusaka is now close to a booming economic area and only two new cases have been reported in the past two decades. One health worker in each village was involved in the project. All suspected konzo cases in each village were identified by community leaders and examined by Dr Nhassico to assess their gait, reflexes and the presence of ankle clonus. History of onset was also recorded. They were confirmed as konzo cases if they met the WHO criteria (World Health Organisation, 1996). All konzo cases were encouraged to do basic rehabilitation exercises.

Fifty women in each village were surveyed with regard to the various types of food they had eaten the previous day. Fifty urine samples were obtained from school children in each village with the oral consent of community leaders and school teachers and were analyzed for thiocyanate. Cassava flour samples (42–50 from each village) were obtained from families and were analyzed for total cyanide. The team trained 30 senior women from each village to use the wetting method and they in turn trained 5–8 other women in the village. This resulted in the training of 150–240 women in each village, with a lower number being trained in Cava. To facilitate use of the wetting method, laminated posters in Portuguese describing the wetting method were distributed to 190–270 women in each village (Bradbury et al. 2011) and an equal number of bowls, knives and mats.

Subsequently over the period September 2012–March 2013 Ms Majonda's team from Nampula made three monitoring visits to the three villages to encourage the women to keep using the wetting method and in each village visited 25–30 households. In March 2013 the second visit of the full team was made with a further check on the use of the wetting method by the women, urinary thiocyanate analyses made on 50 samples of urine from school children in each village and cyanide analyses made on cassava flour samples just before they were used to make the daily porridge. This was followed by three monitoring visits from Ms Majonda's team. In September 2013 the third visit of the full team was made to the three villages and urinary thiocyanate and cassava flour analyses made. This was followed by two monitoring visits to encourage women to keep using the wetting method. The fourth and final visit of the full team to collect flour and urine samples as before was in August 2014.

### Urinary thiocyanate analysis

About 50 urine samples were collected from school age children in each village and these samples were analyzed using the simple picrate thiocyanate kit D1 (Haque and Bradbury 1999) http://biology.anu.edu.au/hosted_sites/CCDN/, which contains a color chart with 10 shades of color from yellow to brown that correspond to 0 to 1720 μmol thiocyanate/L.

### Flour cyanide analysis

Samples of cassava flour (40–50) were collected from households in each village before teaching the wetting method and at subsequent visits just before they were used to make nchima. Total cyanide analyses were made using kit B2 (Egan et al. 1998; Bradbury et al. 1999). http://biology.anu.edu.au/hosted_sites/CCDN/.

A color chart was used with 10 shades of color from yellow to brown corresponding to 0–800 mg HCN equivalents/kg cassava flour (ppm).

## Results and Discussion

**Figure 2:**
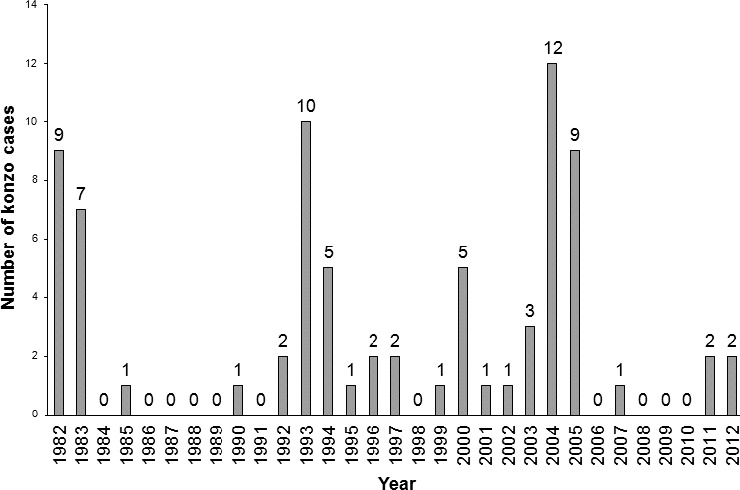
Annual distribution of onset of konzo cases in the three Nampula villages.

In Table 1 is given the number of konzo cases in each village and the percentage konzo prevalences calculated from the population data. There are 77 konzo cases with a konzo prevalence ranging from 0.58% in Mujocojo to 2.9% in Cava. The annual distribution of konzo cases given in Figure 2 shows that the konzo incidence since the last drought in 2005 is low, with one case in 2007 from Cava and two cases in 2011 (one from Cava and one from Mujocojo) and two cases from Mujocojo in 2012. These are cases of “persistent” or “endemic” konzo (Ernesto et al. 2002) that occur in years of adequate rainfall, but in drought years these areas are at risk of konzo epidemics. In Figure 2, taking into account the passage of time and some unreliability in remembering the year of onset, the peaks in 1982–3 are due to severe drought in Acordos de Lusaka and Cava, (Ministry of Health Mozambique, 1984) in 1993–4 due to war and its aftermath in Mujocojo, in 2000 probably due to a low rainfall period in 1998–9, (Cardoso et al. 2005) and in 2004–5 due to a drought in Cava. Because there has been a period of adequate rainfall since 2005 there are very few new cases of konzo in these villages and hence much less incentive for village women to remove konzo‐producing cyanogens, than for village women in DRC where many new konzo cases are occurring every year (Banea et al. 2012, 2013, 2014b). These cases are the result of high cyanide intake from inadequately processed cassava flour made from cassava roots soaked for only 1–2 days instead of the 3–4 days that are needed to remove cyanogens.

**Table 1:** Population, number of konzo cases, and % konzo prevalence in Nampula Province villages

Village	Population	Number of konzo cases	% Konzo prevalence
Cava	1654	48	2.9
Acordos de Lusaka	1618	12	0.74
Mujocojo	2918	17	0.58
Total	6190	77	1.2

Table 2 shows the food consumption patterns in the three villages at the beginning of the intervention in September 2012, at the peak of the cassava harvest. As expected there is a very great dependence on cassava flour eaten as a thick porridge (nchima) with 63–92% of families having consumed nchima on the previous day and 14–40% cassava leaves pounded and boiled to remove cyanogens (Bradbnury and Denton 2014). The combined fish, chicken and goat consumption is lower at 34–60% as expected and other components of the diet are much smaller in amount. The diet is very restricted and heavily focused on cassava flour and cassava leaves. In the monitoring visits it was found that only 30–40% of households were using the wetting method in Acordos de Lusaka and Muocojo and a lower percentage in Cava, possibly due to difficulties in implementation.

**Table 2:** The percentage of families that ate a particular food on the previous day.^a^

Food	Percentage of families that ate a particular food on the previous day from		
	Cava	Acordos de Lusaka	Mujocojo
“Nchima” from			
Cassava flour	62	72	92
Fish	38	32	36
“Matapa”^b^	14	40	20
Beans	20	20	2
Maize meal	20	16	0
Chicken	12	2	16
Rice	10	10	2
“Minane”^c^	0	0	14
Goat meat	10	0	0

a Data collected in September 2012 and arranged in descending order.

b “Matapa” consists of pounded and then boiled cassava leaves, cooked with peanuts, and prawns.

c Minane” is a local wild tuber (Cardoso et al. 2004).

The results of the thiocyanate analyses of the urine of school children are shown in Tables 3 and 4. The lower results obtained in March 2013 are seasonal, due to the fact that the peak cassava harvesting season is August‐October and less cassava is being consumed in March (Casadei et al. 1990; Cliff et al. 2011). There is a downward trend from 2012 to 2014 in the mean values for the August‐September results, with the exception of Mujocojo which was lowest in September 2013. A similar trend is found in Table 4, which shows the percentage of children with high urinary thiocyanate levels (>350 μmol/L), who are considered to be at risk of contracting konzo (Banea et al. 2012, 2015a). There is also a downward trend in the mean total cyanide content of cassava flour shown in Table 5 for all villages. At the completion of the intervention there are 0% and 4% of children in Acordos de Lusaka and Mujocojo, respectively, with high urinary thiocyanate levels (Table 4), which is consistent with the results obtained at the conclusion of four previous interventions in DRC (Banea et al. 2012, 2013, 2014b, 2015a) all of which prevented new cases of konzo from occurring. Combined with the reduction in the mean total cyanide content in flour in Acordos de Lusaka and Mujocojo to 17 ppm and 9 ppm, respectively, this is a satisfactory result. The FAO/WHO maximum safe level for cassava flour is 10 ppm (FAO/WHO, 1991).

**Table 3:** Mean thiocyanate content (μmol/L)^a^ of urine of school children in Nampula Province villages before introducing the wetting method in September 2012 and during the intervention

Village	Mean urinary thiocyanate content (μmol/L) in			
	September 2012^b^	March 2013	September 2013	August 2014
Cava	530 (460)	100 (130)	280 (290)	180 (200)
Acordos de Lusaka	200 (140)	130 (180)	140 (180)	60 (50)
Mujocojo	160 (140)	70 (60)	60 (50)	150 (90)

a Standard deviation in brackets.

b Before introduction of the wetting method.

**Table 4:** Percentage of school children in Nampula Province villages with urinary thiocyanate contents of >350 μmol/L

Village	Percentage of children with urinary thiocyanate content of >350 μmol/L			
	September 2012^a^	March 2013	September 2013	August 2014
Cava	52	2	20	17
Acordos de Lusaka	10	4	3	0
Mujocojo	6	0	0	4

a Before introduction of the wetting method.

**Table 5:** Mean total cyanide content (ppm) of cassava flour in the Nampula villages.^a^

Village	Mean total cyanide content (ppm) of cassava flour samples in			
	September 2012^b^	March 2013^c^	September 2013^c^	August 2014^c^
Cava	64 (52)	66 (42)	56 (56)	25 (19)
Acordos de Lusaka	27 (30)	26 (18)	6 (3)	17 (10)
Mujocojo	17 (15)	24 (14)	20 (12)	9 (6)

a Standard deviation in brackets.

b Samples taken before the wetting method was introduced.

c Samples taken just before flour was used to make nchima.

In contrast, the results for Cava are more challenging with 17% of children with high urinary thiocyanate levels of >350 μmol/L at completion of the intervention and a mean total cyanide content in cassava flour of 25 ppm. Nevertheless, these levels show a great improvement for Cava compared with the initial values in September 2012 in Tables 3, 4, 5. In the six villages in the DRC it was found that the percentage of families using the wetting method was inversely proportional to the mean urinary thiocyante content of the children and the percentage of households using the method was 68–94% (Banea et al. 2015a). In Acordos de Lusaka and Mujocojo only 30–40% used the wetting method with a lower percentage in Cava, which explains why the results in Cava were not as good as in the other two villages. The much lower percentage use of the wetting method by the women in Mozambique compared with the women in the DRC (Banea et al. 2015a) results from lack of incentive to use the method, because of the relative absence of new cases of konzo in the Mozambique villages compared with the DRC villages.

## Conclusion

Konzo outbreaks in East Africa occur under drought conditions, due to large increases by the cassava plant in the cyanide content of cassava roots and hence of cassava flour made from these roots (Cardoso et al. 2005) and also due to war, where postharvest processing may be shortened and processed cassava flour stolen (Cliff et al. 2011). The wetting method has been introduced into three villages in Mozambique with previous konzo outbreaks (see Fig. 2). The uptake of the method by households has been 30–40% in Acordos de Lusaka and Mujocojo and less in Cava, compared with 68–94% in the DRC, (Banea et al. 2015a) because there is little incentive for village women to use the method during a period of normal rainfall, when there are few or no new konzo cases. In contrast, konzo is occurring every year on an increasing scale in Kwango District, Bandundu Province, DRC (Okitundu et al. 2014) and hence village women have more readily accepted the wetting method to reduce cyanide intake and prevent konzo (Banea et al. 2012, 2013, 2015a). We believe that the wetting method should be taught in all those areas in Mozambique and Tanzania where konzo has occurred, so that the wetting method will be fully understood and accepted by village women and be used by them to prevent cyanide intoxication and outbreaks of konzo during future droughts.

## Conflict of Interest

None declared.
